# Elementary teachers’ attitudes and beliefs about spatial thinking and mathematics

**DOI:** 10.1186/s41235-020-00221-w

**Published:** 2020-04-16

**Authors:** Heather Burte, Aaron L. Gardony, Allyson Hutton, Holly A. Taylor

**Affiliations:** 1 Texas A&M University, Psychological and Brain Sciences, 4235 TAMU, College Station, TX 77843 USA; 2 Department of Psychology, Tufts University, 490 Boston Avenue, Medford, MA 02155 USA; 3 Think3d!, 3811 Van Ness Street NW, Washington, DC, 20016 USA

**Keywords:** Elementary school teachers, Spatial thinking, Spatial anxiety, Math beliefs, Math anxiety

## Abstract

Considering how spatial thinking connects to Science, Technology, Engineering and Mathematics (STEM) outcomes, recent studies have evaluated how spatial interventions impact elementary students’ math learning. While promising, these interventions tend to overlook other factors affecting math learning; perceptions of math abilities, beliefs about math, and math anxiety can also impact math performance. Additionally, perceptions of spatial skill and spatial anxiety impact spatial performance. This study investigated how elementary teachers’ perceptions of spatial thinking connects with math perceptions. Specifically, we focused on teachers’ attitudes and beliefs around three topics: teaching and learning math, spatial abilities, and spatial thinking in mathematics. We found that lower spatial anxiety related to lower anxiety about teaching math, greater alignment between math beliefs and math standards, and greater efficacy in teaching and learning math. Further, a factor analysis showed one factor that connected stereotypical math thinking with both math and spatial anxiety, and another that connected spatial competencies, teaching and learning math, and spatial thinking within math. To further evaluate spatial thinking in math, we introduced a math categorization and verified it using teachers’ ratings of teaching difficulty, visualization helpfulness, and spatial-thinking involvement. Structural equation models revealed that the level of spatial-thinking categorization was the best model of all three of the teachers’ ratings. Overall, results showed numerous connections between teachers’ attitudes and beliefs about mathematics and spatial thinking. Future intervention studies should consider teachers who are spatial and/or math-anxious, and future research should investigate the role of stereotypical thinking in spatial and math anxiety.

## Significance

The connection between spatial thinking and STEM outcomes has inspired a growing set of studies evaluating spatial interventions for elementary students’ math learning. However, teachers rarely implement these interventions. This is problematic because scaling these interventions for broader implementation necessitates teachers leading them in their classrooms. A growing literature shows that teachers’ math beliefs and anxiety impact their students’ math beliefs, anxiety, and performance. Therefore, it is crucial to know how teachers’ math beliefs and anxiety relates to their spatial-thinking beliefs and anxieties because interventions must be designed with teacher implementation in mind. The current work investigated connections between teachers’ attitudes and beliefs in three areas: mathematics (e.g., anxiety about teaching math), their own spatial abilities (e.g., spatial anxiety/competency), and spatial thinking in mathematics (e.g., competency with the spatial aspects of mathematics). The numerous connections between teachers’ attitudes and beliefs about mathematics and spatial thinking found in this study indicate that spatial interventions targeting math learning should also include extra support for teachers. This is particularly important for teachers with negative perceptions of their spatial skills and/or math-teaching skills.

## Background

Decades of research has established that mathematics and spatial thinking are highly connected (see Mix & Cheng, 2012 for a review), and more broadly, spatial thinking is found throughout Science, Technology, Engineering, and Mathematics (STEM; Uttal & Cohen, 2012). While this connection is not fully understood in elementary students, recent correlational studies have established the spatial-math connection in young children. Two longitudinal studies connected young children’s spatial abilities, specifically mental rotation and visuospatial memory, to their spatial and math performance at a later age (Gunderson, Ramirez, Beilock, & Levine, 2012; Lauer & Lourenco, 2016). Two studies using a battery of spatial and math tasks found two domain-specific factors (i.e., spatial and math) that were highly correlated and consistent across three elementary school-age groups (Mix et al., 2016, 2017). These studies highlight that developing spatial-thinking skills relates to developing math skills. This connection is currently being leveraged by researchers to create and test spatial interventions aimed at improving elementary students’ math learning.

Connections between spatial thinking and math learning have contributed to a growing set of studies addressing the effectiveness of spatial interventions. Mental rotation training improved elementary students’ mental rotation and missing-term problem performance (Cheng & Mix, 2014). Relatedly, online mental rotation training improved mental rotation and calculation problem performance (Cheung, Sung, & Lourenco, 2019). Spatial training programs covering a broad range of spatial-thinking skills (spatial visualization, mental rotation, and spatial orientation) led to broader spatial-thinking gains along with gains in geometry (Lowrie, Logan, & Ramful, 2017). One potentially effective spatial training program involves origami. Three studies that have used origami to train spatial visualization skills found improvements on spatial visualization and calculation problems amongst students with mathematical difficulties (Krisztián, Bernáth, Gombos, & Vereczkei, 2015). Two other studies found that origami and paper engineering training lead to spatial visualization improvements (Taylor & Hutton, 2013) and improvements on select math problems (Burte, Gardony, Hutton, & Taylor, 2017). Overall, mounting evidence suggests that spatial interventions in elementary school contribute to positive spatial thinking tasks and math problem gains.

While teachers rarely implemented the interventions described in the previously studies, to scale these interventions for broader implementation teachers will need to lead them in their classrooms. Two potential issues emerge with teachers leading spatial interventions for math learning. These relate specifically to the teachers and include: (1) math anxiety and perceptions of math, and (2) spatial anxiety and perceptions of spatial thinking. Mathematics and spatial thinking can cause some people considerable anxiety, and anxiety can impact performance on math and spatial tasks. If spatial interventions for math learning are to be effective outside the laboratory, the field will need to know how teachers’ perceptions of and anxiety about math and spatial thinking are connected. Understanding how perceptions of and anxiety about math and spatial thinking are connected will also connect separate sets of literature. The math-spatial literature has never been connected to the math-anxiety literature, despite the clear overlap.

The math-anxiety literature has shown that teachers’ anxiety and perceptions of math impact their teaching and ultimately their students’ math performance. Elementary school teachers routinely face the challenge of having to teach subjects that they might not like because they teach most subjects their students learn. This contrasts with secondary and post-secondary teachers who tend to specialize within a subject area. Just like their students, teachers prefer particular subjects; those preferences can impact their teaching efficacy. For instance, based on a love of literature a teacher may develop engaging reading assignments while a distaste for math may result in tedious math activities. Teachers’ preferences for (and anxieties towards) particular subjects, along with their knowledge of and attitudes toward those subjects impacts their teaching (e.g., Foley et al., 2017). What drives negative attitudes towards math in teachers? How do those attitudes impact their students?

Negative attitudes towards mathematics, including math anxiety (Harper & Daane, 1998), are not uncommon amongst pre-service and in-service teachers (Cornell, 1999). These negative attitudes often originate from personal experiences as students (Bekdemir, 2010; Harper & Daane, 1998), including poor pedagogical practices (Cornell, 1999). Negative attitudes from childhood can carry into college and pre-service education, leading teachers to avoid math courses beyond the minimum requirements (Brady & Bowd, 2005). Given that few teachers major or minor in mathematics (Ingersoll, 1999) and pre-service training sometimes inadequately prepares for teaching elementary and secondary math (Ball, 1990), teachers can suffer from inadequate math content knowledge (Ma, 1999) and inadequate “mathematical knowledge for teaching” (Ball, Hill, & Bass, 2005). Ultimately, teachers’ beliefs about math and their mathematical knowledge for teaching contribute to their teaching effectiveness (Hill et al., 2008) and their students’ math achievement (Hill, Rowan, & Ball, 2005). This can set up a vicious cycle of negative attitudes towards mathematics.

In summary, a robust literature shows that spatial thinking is connected to mathematics. A separate literature shows how teachers’ math anxiety and attitudes about math impact their students’ math learning. These two literatures have not yet come together to understand how teachers’ spatial-thinking attitudes and abilities might impact their math-teaching attitudes and efficacy. Yet, existing work suggests bringing them together could be informative. Similar to math anxiety, spatial anxiety is not uncommon and more so affects women (Lawton, 1994), who comprise the majority of elementary school teachers. Further, teachers’ spatial anxiety impedes students’ spatial learning (Gunderson, Ramirez, Beilock, & Levine, 2013). Finally, with the increasing examination of spatial interventions for elementary students’ math learning, it is critical that the field knows how elementary teachers’ perceptions of spatial thinking relates to math perceptions. The current research investigates three connections between teachers’ attitudes/beliefs: (1) anxiety for teaching math and beliefs about math, (2) self-reported spatial abilities, and (3) experience with spatial aspects of math. Secondarily, we explore a math categorization that may help identify where teachers see spatial thinking in math.

### Math anxiety

Math anxiety has consequences for the individual experiencing that anxiety. It results in negative perceptions of ability and poor performance (Vukovic, Kieffer, Bailey, & Harari, 2013), can lead to math avoidance, and ultimately, reduced career prospects (Ashcraft, 2002). Math anxiety can extend beyond the individual, particularly for teachers. A teachers’ anxiety about teaching math relates to lower student math achievement (Hadley & Dorward, 2011), potentially by reducing teaching efficacy (Bursal & Paznokas, 2006) and/or limiting content knowledge, as evidenced by reduced alignment with the National Council of Teachers of Mathematics standards (National Council of Teachers of Mathematics (NCTM), 2000; Swars, Daane, & Giesen, 2006). The NCTM has advocated for reforms in mathematics education and has issued standards outline underlying assumptions about math and math education (e.g., learning is active, conceptually oriented math education, numerous methods can produce the correct answer). Pre-service teachers’ beliefs about math and math education can shift towards being more aligned with the NCTM standards as they take coursework on math and math education (Hart, 2002). Further, teachers who are math anxious and/or hold gendered math attitudes can pass those attitudes onto students (Gunderson, Ramirez, Levine, & Beilock, 2012). Math-anxious female teachers influence female students’ negative math attitudes by reinforcing a “math is for boys” stereotype; this then contributes to female students’ poor math performance (Beilock, Gunderson, Ramirez, & Levine, 2010). Based on this literature, we predict that lower anxiety about teaching mathematics will relate to greater perceived efficacy in teaching and learning math (or “math efficacy”), and greater alignment of beliefs with the NCTM standards. We also investigate whether more years spent teaching math and daily teaching of math is associated with lower math anxiety, greater math efficacy, and greater alignment with the NCTM standards.

### Linking spatial thinking and spatial anxiety to math

Elementary math involves spatial thinking, yet spatial thinking is largely missing from elementary education (National Research Council, 2006). Numerical knowledge can be spatially conceptualized as numbers arranged along a line. In fact, children’s spatial abilities predict improved number line understanding across grades 1 or 2, and children’s early spatial abilities predict later symbolic math abilities, as mediated by number line understanding (Gunderson et al., 2012). However, the relationship between spatial ability and math performance is not always straightforward. In middle school, boys’ – but not girls’ – mental rotation performance predicted math achievement (Ganley & Vasilyeva, 2011). Spatial training programs improve children’s spatial thinking but only improve math performance in a targeted way (e.g., Burte et al., 2017; Cheng & Mix, 2014).

The connection between teachers’ spatial abilities, spatial anxiety, and anxiety in teaching mathematics has not yet been explored, despite research supporting a link. Both spatial thinking and mathematics can induce anxiety and more so in female teachers. Further, spatial abilities and spatial anxiety both predict math anxiety (Ferguson, Maloney, Fugelsang, & Risko, 2015). The male advantage in math performance has been related to spatial ability (Geary, Saults, Liu, & Hoard, 2000), to a mediation effect of male spatial abilities and math self-confidence (Casey, Nuttall, & Pezaris, 1997), and to a mediation effect of spatial imagery abilities (Maloney, Waechter, Risko, & Fugelsang, 2012). In the current research, we predict that greater spatial competency will relate to lower anxiety about teaching math, greater math efficacy, greater alignment with NCTM standards, and more years spent teaching math. The opposite pattern will be found for spatial anxiety.

We also examine the relationship between teachers’ own academic experience with spatial aspects of math (i.e., level of education for last math course taken and academic success in the spatial aspects of that course) and our spatial and mathematics measures. We predict that taking math courses at higher educational levels and greater success with the spatial aspects of that course will relate to greater spatial competency and math efficacy, lower math and spatial anxiety, greater alignment with NCTM standards, and more years spent teaching math.

In a recent study, Mix et al. (2016, 2017) evaluated the latent structure of spatial and mathematical skills in elementary students using a series of exploratory factor analyses. They found that the two domains were highly related and that a battery of spatial and mathematical measures separated into domain-specific factors. While spatial measures tended to group together in one factor and math measures tended to group together in a separate factor, there were a few spatial measures that loaded on the math factor and vice versa. This pattern held for students in Kindergarten, grade 3, and grade 6. Given these results, we predict that math and spatial measures taken by elementary teachers will tend to group into their respective domains.

### Spatial thinking in mathematics

**Fig. 1:**
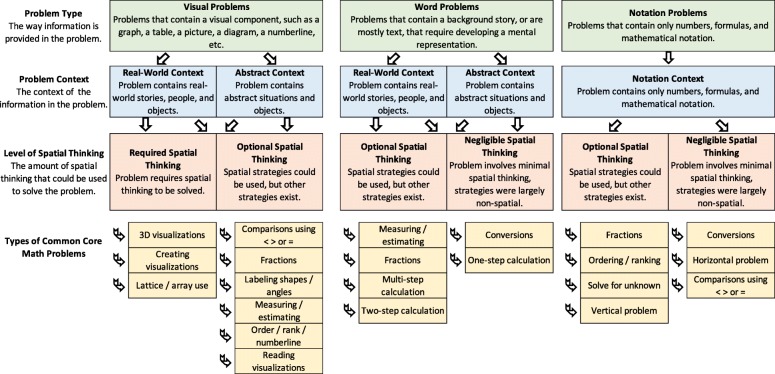
Math categorization including definitions of problem type, problem context, and spatial thinking. Flow chart of how each problem type relates to problem contexts, then how problems contexts relate to spatial thinking, and then types of Common Core Math problems that fit within each grouping

**Fig. 2:**
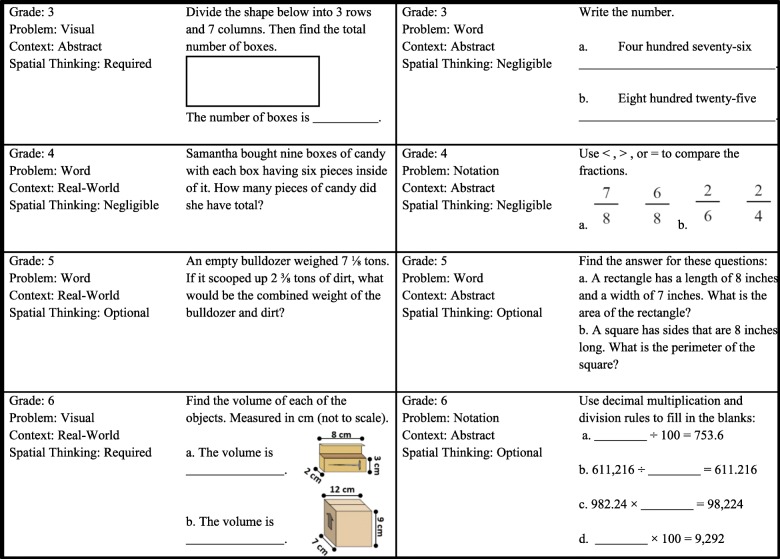
Example Common Core Math problems categorized by problem type, context, and spatial thinking. Sourced from sourced from www.commoncoresheets.com

Secondarily, just because spatial thinking relates to math success does not mean that every math problem engages spatial thinking. To help identify spatial thinking in math, we developed a math categorization based on the following factors: *problem type* (visual, word, notation), *problem context* (abstract, real-world, notation), and *spatial-thinking level* (required, optional, negligible). *Problem type* reflects the ways that math problems present information: visual representations include pictures or diagrams, word representations present problems as stories, and notation representations use mathematical notation. *Problem context* refers to whether the problem uses a real-world, abstract, or mathematical notation context. Finally, *level of spatial thinking* indicates whether problems require, optionally involve, or have negligible spatial thinking. Problems required spatial thinking when they could only be solved using spatial-thinking strategies, problems involved optional spatial thinking when they had both spatial and non-spatial solution strategies, and problems involved negligible spatial thinking when solution strategies were largely non-spatial. While any math problem could be solved using a spatial strategy, we categorized problems that elicited the least amount of spatial thinking or problems that students were unlikely to use a spatial strategy as “negligible.” This is not to suggest that no spatial thinking was used to solve these problems, as even reading is spatial, but just that students were expected to use largely non-spatial strategies (e.g., memorized math facts, simple comparisons). See Fig. 1 for a visualization of this categorization and Fig. 2 for example problems.

We applied our categorization to problems aligned with the *Common Core State Standards for Mathematics* (National Governors Association Center for Best Practices and Council of Chief State School Officers, 2010), although it applies to math problems more generally. As can be seen in Fig. 1, there are interactions between the three factors that can result in the same types of Common Core Math problems being categorized as different levels of spatial thinking due to their problem type and context. For example, a comparison between two numbers (3 ___4) is a notation problem and notation context because only numbers and mathematical notations are involved. Here, spatial thinking involvement is low, so the level of spatial thinking is “negligible.” Another comparison problem might involve figures representing fractions (a picture of a circle showing ½ _____ a picture of a rectangle showing ¾). In this case, the problem is visual because of the figures, the context is abstract because circles and rectangles are used, and the spatial-thinking involvement is more substantial than the number comparison problem because of the need to interpret the figures. Here, spatial thinking is “optional.”

To validate our categorization, teachers rated Common Core problems for perceived teaching difficulty, spatial thinking involved, and helpfulness of visualizations for solving the problem. These ratings will provide insight into ways that math problems involve spatial thinking, along with how teachers perceive and approach teaching a range of math concepts. Different ratings across problem types, problem contexts, and spatial thinking involved would support our categorization. We will also use structural equation modeling to evaluate whether the math problems are best modeled as one factor or as factors based on the math categorization. If the structural equation models indicate that our factors are better models of the math problems than a one-factor model, then the structural equation models will have provided support for our math categorization.

### Current study

The current research assesses how teacher attitudes around mathematics, spatial thinking, and spatial thinking in mathematics relate to each other, testing the following hypotheses:

1. *Attitudes about mathematics*: Teachers’ years spent teaching mathematics, perceived math efficacy, and NCTM alignment will all be positively related. Anxiety about teaching math will be negatively related to those three measures.
2. *Attitudes about mathematics and spatial thinking*: Teachers’ spatial competency will be negatively related to their spatial anxiety. Teachers’ spatial competency will be positively related to their perceived math efficacy and NCTM alignment. Teachers’ spatial anxiety will be positively related to their anxiety about teaching mathematics, and both anxiety measures will be negatively related to those three measures.
3. *Spatial thinking in mathematics*: Level of education of last taken math course will be positively related to the amount of spatial thinking involved in that math course. Teachers’ reported competency with the spatial aspects in that math course will relate positively to the three attitudes about mathematics and spatial-thinking measures and negatively to the spatial and teaching math-anxiety measures.
4. *Exploratory factor analysis of spatial and mathematics measures*: Spatial measures (spatial competency and anxiety) will tend to load on a separate factor from the mathematics measures (years spent teaching mathematics, how often teach math, math efficacy, NCTM alignment, anxiety about teaching math, level of education of last math course, amount of spatial thinking in last math course, and competency with the spatial aspects in last math course). Some crossover of measures may occur, which will provide insights into the connections between teachers’ perceptions of spatial thinking and math.
5. *Math problem categorization*: Ratings of teaching difficulty, spatial-thinking involvement, and visualization helpfulness will differ across the categorizations of problem type, problem context, and level of spatial thinking involved. Visual, real-world, and required spatial-thinking problems will be rated more difficult to teach, requiring more spatial thinking, and more helped by visualizations than word, abstract, and optional spatial-thinking problems. Word, abstract, and optional spatial thinking problems will be rated more difficult to teach, requiring more spatial thinking, and more helped by visualizations than notation and negligible spatial thinking problems.
6. *Structural equation models of the math categorization*: Separate models for each rating (teaching difficulty, spatial-thinking involvement, and visualization helpfulness) split by each math category (problem type, problem context, and level of spatial-thinking involvement) will be run. The three-factor models, that align with the math categorization, will more accurately represent the data compared to one factor models of all the math problems together, thereby providing support for the math categorization.

## Method

### Ethics and consent

This research was approved by the Tufts University Social, Behavioral, and Educational Research Institutional Review Board (protocol #1506030). Participants read through a consent form online and indicated their consent before participating in the study.

### Participants

We recruited elementary teachers through multiple sources: locally through email and Facebook, and more broadly through a Qualtrics panel (Qualtrics, Provo, UT, USA). Teachers from New England schools were contacted by email (*N* = 48), teachers affiliated with Tufts’ Center for Engineering Education and Outreach were contacted through a Facebook post (*N* = 12), and Qualtrics contacted a teacher cohort (*N* = 123). Only teachers who completed the entire online Qualtrics survey were included in the analyses (*N* = 173). Of those teachers (ages 19–70 years, *M*_*age*_ = 42.59; *SD*_*age*_ = 11.59), 12 were male, 161 were female, and they had between zero and 48 years of teaching experience (*M* = 13.98; *SD* = 10.09).

### Materials and procedure

Teachers completed the following questionnaires in order: demographics, educational and teaching background (Tables 1 and 2), spatial competency and anxiety scales, anxiety about teaching mathematics scale, mathematics belief instrument (Parts B and C), math categorization ratings, and math education questions. See Table 3 for number of questions, example questions, and possible responses for each measure.

**Table 1:** Percent of teachers by educational background

Educational background	Percentage of teachers
Associate’s degree	8.7%
Bachelor’s degree	37.6%
Master’s degree	42.8%
PhD	11.0%
Hold a teaching license	90.7%
Licensed with degree	91.1%
Elementary education licensure	65.9%

**Table 2:** Percentage of teachers by grade(s) taught in the previous year, subject(s) taught daily in previous year, and mean years teaching each subject

Grade(s) taught		Daily teaching of subject(s)		Mean years teaching subject(s)	
Kindergarten	18.1%	Language arts	72.3%	Language arts	12.69
1st grade	32.9%	Social studies	29.5%	Social studies	11.88
2nd grade	30.6%	Mathematics	74.0%	Mathematics	12.39
3rd grade	32.4%	Science	28.9%	Science	11.57
4th grade	38.7%	Foreign languages	3.5%	Foreign languages	1.28
5th grade	32.9%	Art	5.2%	Art	3.31
6th grade	17.9%	Music	4.6%	Music	2.99
Administration	6.9%	Physical education	5.8%	Physical education	2.41

Note: Percentages sum to > 100% because teachers taught multiple grades and subjects

**Table 3:** Measures used along with number of questions, and example question, and range of responses

Measures	Questions	Example question	Responses
Spatial competency	15	How would you do? Finding your way to an appointment in an unfamiliar areas of a city or town	1 = Terribly 5 = Very well
(8 navigation-related from Lawton, 1994)			
(7 everyday spatial tasks)			
Spatial anxiety	15	How would you feel? Following visual directions to put “assembly required” furniture (e.g., Ikea) together	1 = Not at all anxious 5 = Very anxious
(8 navigation-related from Lawton, 1994)			
(7 everyday spatial tasks)			
Anxiety about teaching mathematics	12	Rate how much anxiety you experience: Looking through the pages in your math series teacher’s manual	1 = Low anxiety 5 = High anxiety
(Hadley & Dorward, 2011)			
Mathematics belief instrument, Part B or “NCTM alignment” (Hart, 2002)	12	Respond with your beliefs about the truthfulness of the statement: Some people are good at mathematics and some aren’t	1 = True 4 = False
Mathematics belief instrument, Part C or “Math efficacy” (Hart, 2002)	2	Respond with your beliefs about the truthfulness of the statement: I am very good at (teaching/learning) mathematics	1 = True 4 = False
Most recent math course	1	Recall the last course you took in math and when you completed it	1 = High school 4 = Graduate school
Spatial-thinking involvement	1	How much did your last math course involve spatial thinking?	1 = No spatial thinking 4 = Substantial amount
Competency in spatial aspects	1	Rate how you handled the spatial aspects of your last math course	1 = I failed 4 = I excelled
Teaching difficulty	12	How difficult is it for you to teach the math concept(s) involved in this problem?	1 = Very difficult 5 = Very easy
Visualization helpfulness	12	How helpful do you think creating and/or using visualization(s) could be in answering this math problem?	1 = Not at all helpful 5 = Very helpful
Spatial-thinking involvement	12	How much spatial thinking do you think could be involved in answering this math problem?	1 = No spatial thinking 4 = Substantial amount

*NCTM* National Council of Teachers of Mathematics standards

#### Demographics, educational and teaching background

This questionnaire asked teachers about the following: age, gender, highest education level, teaching licensure method, licensure subject, years as an educator, current school, grade(s) taught in the previous year, frequency teaching particular academic subjects in the previous year, and years teaching particular academic subjects overall.

#### Spatial competency and anxiety scales

Teachers read 15 descriptions of everyday spatial tasks and rated their competency and anxiety with each. Eight of the descriptions were of large-scale spatial tasks (sourced from Lawton, 1994), and seven of the descriptions were of small-scale spatial tasks. Cronbach’s alpha for all 15 competency items was .87 and for all 15 anxiety items was .91, which indicate good to high reliability.

#### Anxiety about teaching mathematics scale

Teachers read 12 scenarios about preparing to teach and teaching mathematics and rated the anxiety each would induce (Hadley & Dorward, 2011). Cronbach’s alpha for the 12 scenarios was .93, indicating high reliability.

#### Mathematics belief instrument, Parts B and C

The Mathematics Belief Instrument consists of 12 (Part B) and two (Part C) belief statements about mathematics (Hart, 2002). In Part B, teachers rate the truthfulness of mathematical belief statements that are aligned or misaligned with the National Council of Teachers of Mathematics (NCTM) standards. The NCTM standards outline underlying assumptions about math and math education (e.g., learning is active, conceptually oriented math education, numerous methods can produce the correct answer). Teachers’ average score on Part B indicates how well their beliefs about math and math education aligns with the NCTM standards. In Part C, teachers rates beliefs about efficacy with teaching and learning mathematics. Cronbach’s alpha for the 12 items in Part B was .83 and for the two items in Part C was .79, both of which indicate good reliability.

#### Math categorization

Teachers rated math problems for the level of spatial thinking involved and the helpfulness of visualizations in solving them. They received definitions of spatial thinking and visualizations to ensure similar conceptualizations when making ratings. Teachers noted the grade level (grades 3, 4, 5, and 6) with which they were most familiar and then received 12 Common Core Math questions associated with that grade (Fig. 1), sourced from www.commoncoresheets.com. For each math question, they made three ratings: difficulty in teaching the concept(s), spatial thinking that could be involved to solve the problem, and helpfulness of creating and/or using visualizations in solving the problem. For each math category, two to four problems were presented to teachers, for a total of 12 problems. Cronbach’s alpha for the 12 difficulty in teaching ratings was .89, for the 12 spatial-thinking involvement ratings was .85, and for the 12 helpfulness of visualizations was .84, all indicating good reliability.

#### Math education

Teachers were asked to recall the most recent mathematics course that they had completed. They provided the course name/topic, level of schooling when course was taken, rated the overall level of spatial thinking involved in the course (using the same definition of spatial thinking and same spatial-thinking involvement rating as used in the math categorization), and rated their perceived competency in handling those spatial-thinking aspects.

## Results

### Anxiety about teaching mathematics scale

Item ratings were summed for an overall score (60 = highest anxiety; 12 = lowest). Teachers had relatively low anxiety about teaching math (*M* = 28.24, *SD* = 10.71) and anxiety reduced with years spent teaching math, *r*(169) = −.28, *p* < .001.

### Mathematics belief instrument

For Part B, scores near 4 represented a greater alignment between a teacher’s beliefs about math and math education with NCTM standards or “NCTM alignment” (reverse-coding inconsistent items). Teachers’ beliefs aligned moderately with NCTM standards (*M* = 2.99, *SD* = 0.50). Teachers with more years of math-teaching experience, *r*(169) = .16, *p* < .05, and less math-teaching anxiety, *r*(171) = −.40, *p* < .001, had stronger NCTM alignment.

For Part C, scores near 4 represented greater “math efficacy,” i.e., efficacy in teaching and learning math (reverse-coding inconsistent items). Teachers believed that they were moderately effective at teaching and learning math (*M* = 2.94, *SD* = 0.79), and this marginally related to their NCTM alignment, *r*(171) = .14, *p* = .06. Math efficacy was also negatively correlated with math-teaching anxiety, *r*(171) = −.43, *p* < .001, and positively correlated with years teaching math, *r*(169) = .16, *p* < .05.

### Spatial competency and anxiety

The spatial competency and anxiety scales both contained eight statements focusing on navigation tasks (Lawton, 1994), to which we added seven statements reflecting non-navigation spatial tasks (e.g., assembling Ikea furniture or packing the trunk of a car). This range of everyday spatial tasks increases the generalizability of the spatial competency/anxiety scale (Hegarty, Montello, Richardson, Ishikawa, & Lovelace, 2006). The 15 ratings were averaged to produce *spatial competency* and *spatial anxiety* scores (5 represents high competency or anxiety, respectively). Overall, teachers rated themselves as moderately spatially competent (*M* = 3.54; *SD* = 0.60) and only slightly spatially anxious (*M* = 2.26; *SD* = 0.71); these measures were negatively correlated, *r*(171) = −.32, *p* < .001.

Math-anxious teachers were also spatially anxious, *r*(171) = .51, *p* < .001, and felt less competent at spatial task, *r*(171) = −.17, *p* < .05. NCTM alignment negatively correlated with spatial anxiety, *r*(171) = −.32, *p* < .001, but did not correlate with spatial competency, *r*(171) = −.06, *p* = .44. Math efficacy positively correlated with spatial competency, *r*(171) = .36, *p* < .001, and negatively correlated with spatial anxiety, *r*(171) = −.22, *p* < .01.

### Math education level and experience

A third of teachers took their most recent math course as an undergraduate or graduate student (54.9%) and indicated that this course involved substantial spatial thinking (54.3%). Many felt that they either did well (46.8%) or excelled (21.4%) at the course’s spatial aspects. Despite the range of different teaching techniques, course topics, and subject matter of mathematics courses that teachers last took, teachers reported that higher-level courses tended to have more spatial thinking, *r*(105) = .36, *p* < .001. Teachers were more likely to report competently handling the spatial-thinking aspects of higher-level courses, *r*(103) = .28, *p* < .01.

Advanced coursework appears to support math teaching. Mathematics’ education level positively correlated with NCTM alignment, *r*(107) = .43, *p* < .001, efficacy teaching and learning math, *r*(107) = .21, *p* < .05, and with years teaching math, *r*(105) = .23, *p* < .05. Math education level negatively correlated with spatial anxiety, *r*(107) = −.22, *p* < .02.

Teachers’ perceived competency with spatial aspects of their last math course increases their comfort with spatial thinking in a math context. Perceived competency with spatial aspects of their last math course positively correlated with spatial competency, *r*(153) = .23, *p* < .01, and negatively correlated with both math-teaching anxiety, *r*(153) = −.29, *p* < .001 and spatial anxiety, *r*(153) = −.24, *p* < .01. Spatial competency within math also positively correlated with efficacy in teaching and learning mathematics, *r*(153) = .36, *p* < .001.

Teaching math more frequently provides important math experience. Those who taught daily (74.0%) had lower math-teaching anxiety (*M* = 26.45, *SD* = 9.59), *t*(168) = 4.43, *p* < .001, and higher NCTM alignment (*M* = 3.06, *SD* = 0.49), *t*(168) = − 3.26, *p* < .01, compared to teachers who taught math less frequently (26.0%; math-teaching anxiety: *M* = 34.43, *SD* = 11.58; NCTM alignment *M* = 2.77, *SD* = 0.48). Teachers engaging in daily math teaching reported lower spatial anxiety (*M* = 2.17, *SD* = 0.71) compared to those who taught less frequently (*M* = 2.56, *SD* = 0.66), *t*(168) = 3.16, *p* < .01. Not unexpectedly, amongst those who teach math daily, years teaching math related to lower math-teaching anxiety, *r*(126) = −.24, *p* < .01, and higher math efficacy, *r*(126) = .19, *p* < .05. These relationships were not found in teachers who taught math less frequently.

### Exploratory factor analysis of spatial and mathematics measures

The following measures were entered into an exploratory factor analysis with varimax rotation to determine whether the spatial and math measures loaded on the same or separate underlying factors: spatial competency, spatial anxiety, NCTM alignment, teaching/learning math efficacy, years teaching math, teaching math frequency, education level if last math course, spatial thinking involved in last math course, and competency with the spatial thinking in the last math course. We only included the 103 teachers who completed all of these measures into this analysis, which might produce slightly different correlations than we reported in previous sections. The factor analysis was deemed suitable using all the measures based on the following indicators: (1) each measure significantly correlated (adjusted for multiple comparisons) with at least one other measure at the .30 level (see the correlation matrix in Table 4); (2) the Kaiser-Meyer-Olkin measure of sample adequacy was .66, which is above the recommended value of .60; (3) Bartlett’s test of sphericity was significant, *X*^*2*^(45) = 266.03, *p* < .001; and, (4) the communalities were all well above .30, indicating that each item shared common variables with other items (Table 5). The first four factors together represent 68.4% of the available variance, broken down into 33.5%, 14.9%, 10.6%, and 9.5%, respectively. All four eigenvalues were above or near 1 (3.35, 1.49, 1.06 and 0.95, respectively), and the scree plot showed a reduced slope after the four factor. All measures exceeded a minimum criterion of having a primary factor loading of .40 or above, so all measures were retained.

**Table 4:** Correlation coefficients for the exploratory factor analysis

	Spatial anxiety	Spatial competency	Often teach math	Years teach math	Teach math anxiety	NCTM alignment	Math efficacy	Math course level	Math course spatial	Math course spatial competency
*M*	2.24/5	3.56/5	1.73/6	13.17/48	28.99/58	3.10/4	2.96/4	2.83/5	3.60/5	3.14/5
*SD*	0.69/5	0.61/5	1.69/6	10.28/48	10.76/58	0.48/4	0.83/4	0.99/5	0.76/5	0.82/5
Spatial anxiety	–	− .41 ***	.20 ns	.00 ns	.47 ***	− .40 ***	− .27 ns	− .26 ns	.09 ns	− .36 **
Spatial competency		–	− .07 ns	.11 ns	− .19 ns	.11 ns	.39 ***	.08 ns	− .12 ns	.25 ns
Often teach math			–	− .38 **	.37 **	− .27 ns	− .36 **	− .25 ns	− .15 ns	− .14 ns
Years teach math				–	− .28 ns	.27 ns	.23 ns	.23 ns	.09 ns	.13 ns
Teach math anxiety					–	− .55 ***	− .52 ***	− .24 ns	.00 ns	− .44 ***
NCTM alignment						–	.23 ns	.43 ***	− .05 ns	.31 *
Math efficacy							–	.23 ns	− .11 ns	.49 ***
Math course Level								–	.31 *	.26 ns
Math course Spatial									–	.02 ns
Math course Spatial competency										–

Adjusted for multiple comparisons: ***p < .001; **p < .01; *p < .05; *ns* not significant

*NCTM* National Council of Teachers of Mathematics standards

**Table 5:** Factor loadings and communalities from the exploratory factor analysis

	Factor 1	Factor 2	Factor 3	Factor 4	Communalities
Spatial anxiety	−.44				.84
Spatial competency				.66	.93
Often teach math			.57		.88
Years teach math			−.68		.88
Teach math anxiety	−.45				.75
NCTM alignment	.67				.84
Math efficacy				.53	.80
Math course level		.58			.76
Math course spatial		.78			.87
Math course spatial competency				.42	.82

Factor loadings under .40 were suppressed

Overall, these factors reveal that beliefs and attitudes towards math and spatial thinking are not separate – they are highly intertwined. The first factor indicates that stereotypical thinking about math is related to anxiety about both teaching math and doing spatial tasks. The second factor reveals that teachers perceive math courses at higher educational levels to involve more spatial thinking. The third factor indicates that teachers with more years teaching math are also those teachers who tend to teach math daily (1 = daily; 5 = rarely). The fourth factor connects self-perceptions of competency: competency in spatial tasks, teaching and learning math (or “efficacy”), and in the spatial aspects of their last math course. The first, second, and fourth factors all include a combination of spatial and math measures, which indicates that spatial thinking and mathematical reasoning are highly connected for elementary school teachers.

### Math categorization

For math categorization ratings, each teacher indicated the grade level (3, 4, 5, 6) with which they had greatest familiarity. They then received 12 math problems from that grade. Two to four problems fit each math categorization factor. Thus, any trends after averaging over the four grades and two to four problems would be more indicative of the categorization scheme than the particular qualities of each problem.

**Fig. 3:**
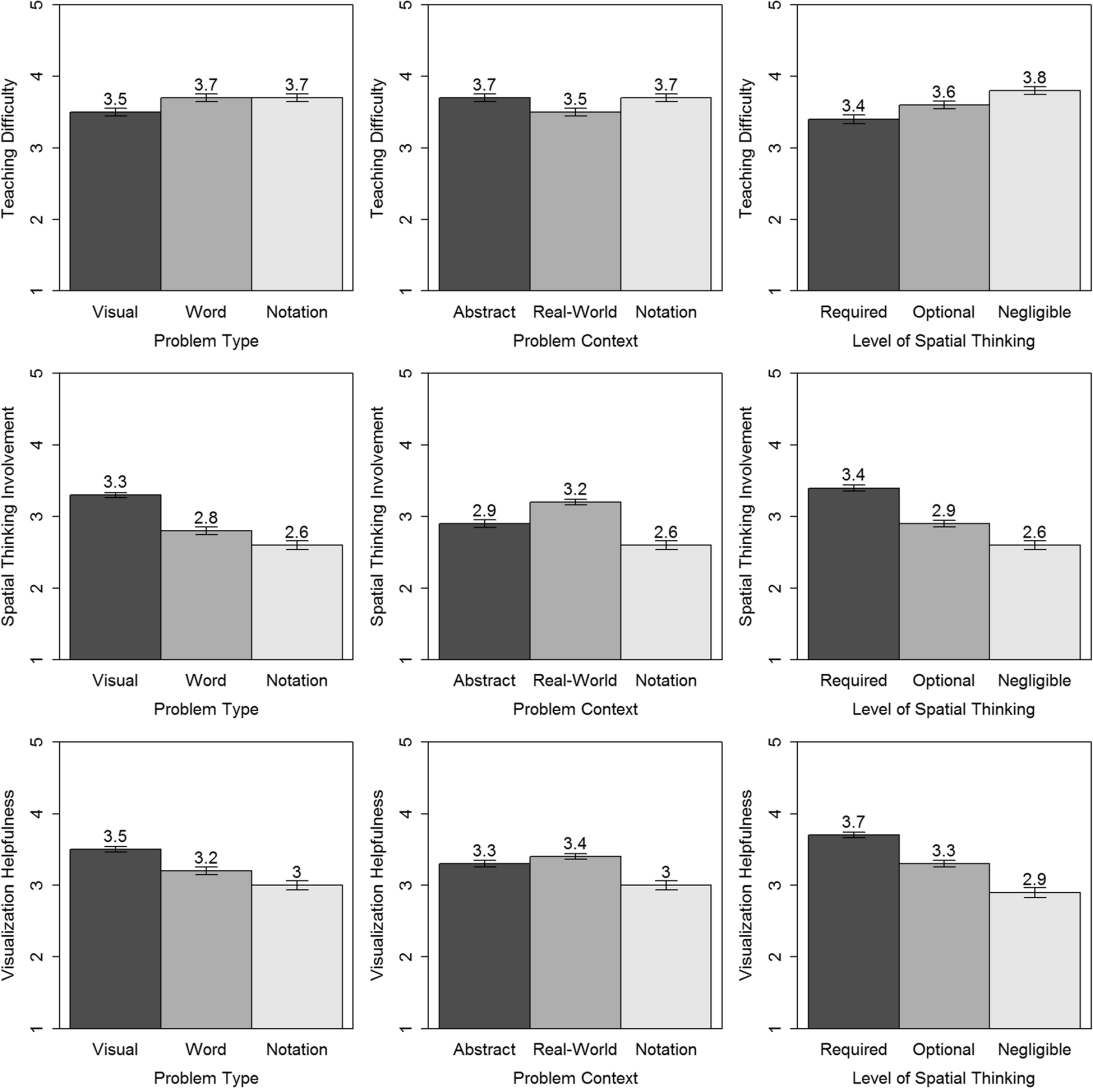
Math categorization by problem type (left column), problem context (center column), and level of spatial thinking (right column). Each by ratings of teaching difficulty (top row), spatial thinking involved (middle row), and helpfulness of visualizations (bottom row). Each graph contains mean values and error bars using the standard error of the mean

Math problems differ substantively, impacting how they can be both solved and taught. Our math categorization aimed to evaluate these differences, including spatial thinking. Analyzes involved 3 (problem type: visual, word, notation) × 3 (problem context: abstract, real-world, notation) × 3 (spatial-thinking level: required, optional, negligible) within-subject analyses of variance (ANOVAs), applied to teaching difficulty, spatial thinking involved, and helpfulness of creating/using visualizations ratings (Fig. 3). All *p* values for significant pairwise comparisons were less than .05.

#### Teaching difficulty

Teaching difficulty ratings mapped onto our categorization. Problem type affected teaching difficulty ratings, *F*(2, 344) = 12.01, *p* < .001, with visual problems rated as harder to teach than word and notation problems, which did not differ. Problem contexts also affected teaching difficulty ratings, *F*(2, 344) = 17.73, *p* < .001. Real-world problems were rated as harder to teach than abstract and notation context problems, which did not differ. Level of spatial thinking also impacted teaching difficulty ratings, *F*(2, 344) = 29.34, *p* < .001. Teaching difficulty ratings increased as the problems had more spatial thinking involved.

#### Visualization helpfulness

Visualization helpfulness ratings also mapped onto our math categorization. Problem type impacted visualization helpfulness, *F*(2, 344) = 66.73, *p* < .001. Teachers rated visualizations as most helpful for visual problems, next for word problems, and least for notation problems. Problem context affected visualization helpfulness ratings, *F*(2, 344) = 50.29, *p* < .001. Teachers rated visualizations as being most helpful for real-world contexts, next for abstract contexts, and least for notation contexts. The level of spatial thinking involved also impacted visualization helpfulness ratings, *F*(2, 342) = 90.23, *p* < .001. Teachers rated visualizations as being more helpful the more it involved spatial thinking. The pairwise comparisons were significant for all three ANOVAs.

#### Spatial-thinking involvement

Ratings of spatial-thinking involvement mapped onto our math categorization. Problem type impacted spatial-thinking ratings, *F*(2, 344) = 120.16. Teachers rated visual problems as involving more spatial thinking than either word or notation problems, and rated word problems as having more spatial thinking than notation problems. Problem context also affected spatial-thinking ratings, *F*(2, 344) = 84.00, *p* < .001. Teachers gave real-world contexts the highest spatial-thinking ratings followed by abstract contexts and then notation contexts. All pairwise comparisons were significant. Ratings of spatial thinking involved matched our categorization of level of spatial thinking involved, *F*(2, 342) = 138.27, *p* < .001. The more the categorization identified problems as using spatial thinking the more teachers rated them as requiring spatial thinking. All pairwise comparisons were significant.

### Structural equation models of the math categorization

In order to investigate whether the math categorization accurately represents underlying constructs, we ran four structural equation models for each of the three ratings that teachers made. The first model (or “one-factor model”) contained all of the math problems under one underlying construct. The second model split the problems by problem type, the third split by problem context, and the fourth split by level of spatial thinking. We then compared the first model to the other models to determine which model most accurately represents the data. If the one-factor model best explains the data, then we will have evidence against our math categorization. However, if the second, third, and/or fourth models best explain the data, then we will have evidence for our math categorization and evidence of which categories (problem type, problem context, and level of spatial thinking) best explain the data. We compared the models using goodness of fit, badness of fit, and fit indices (Table 6).

**Table 6:** Goodness of fit, badness of fit, and fit indices for each structural equation model

	One factor	Problem type (reclassified)	Problem context (reclassified)	Level of spatial thinking
Teaching difficulty				
CFI	.91	.91	.90	.93
TLI	.88	.88	.88	.90
RMSEA	.09	.09	.09	.08
SRMR	.05	.06	.06	.05
AIC	4583	4585	4583	4570
ECVI	1.06	1.06	1.07	0.98
Spatial-thinking involvement				
CFI	.92	.92	.91	.93
TLI	.90	.89	.89	.91
RMSEA	.07	.08	.08	.07
SRMR	.06	.06	.06	.06
AIC	5108	5110	5110	5100
ECVI	0.89	0.91	0.90	0.85
Visualization helpfulness				
CFI	.92	.92	.91	.97
TLI	.90	.89	.89	.96
RMSEA	.07	.07	.07	.05
SRMR	.07	.06	.07	.05
AIC	4821	4825	4826	4800
ECVI	0.85	0.87	0.88	0.72

*CFI* Comparative Fit Index, *TLI* Tucker Lewis Index, *RMSEA* Root Mean Square Error of Approximation, *SRMR* Standardized Root Mean Square Residual, *AIC* Akaike Information Criterion, *ECVI* Expected Cross Validation Index

In term of goodness of fit indices, the Comparative Fit Index (CFI) and Tucker Lewis Index (TLI) indicate how well the data fit the specified model and values above .90 are best. For badness of fit indices, Root Mean Square Error of Approximation (RMSEA) and Standardized Root Mean Square Residual (SRMR) indicate a misfit in the specified model and values below .10 are best. Two fit indices, the Akaike Information Criterion (AIC) and Expected Cross Validation Index (ECVI), were used to compare the models. Smaller values on both fit indices indicate better fit. Finally, we used chi-squared tests to evaluate significant differences between the models.

**Fig. 4:**
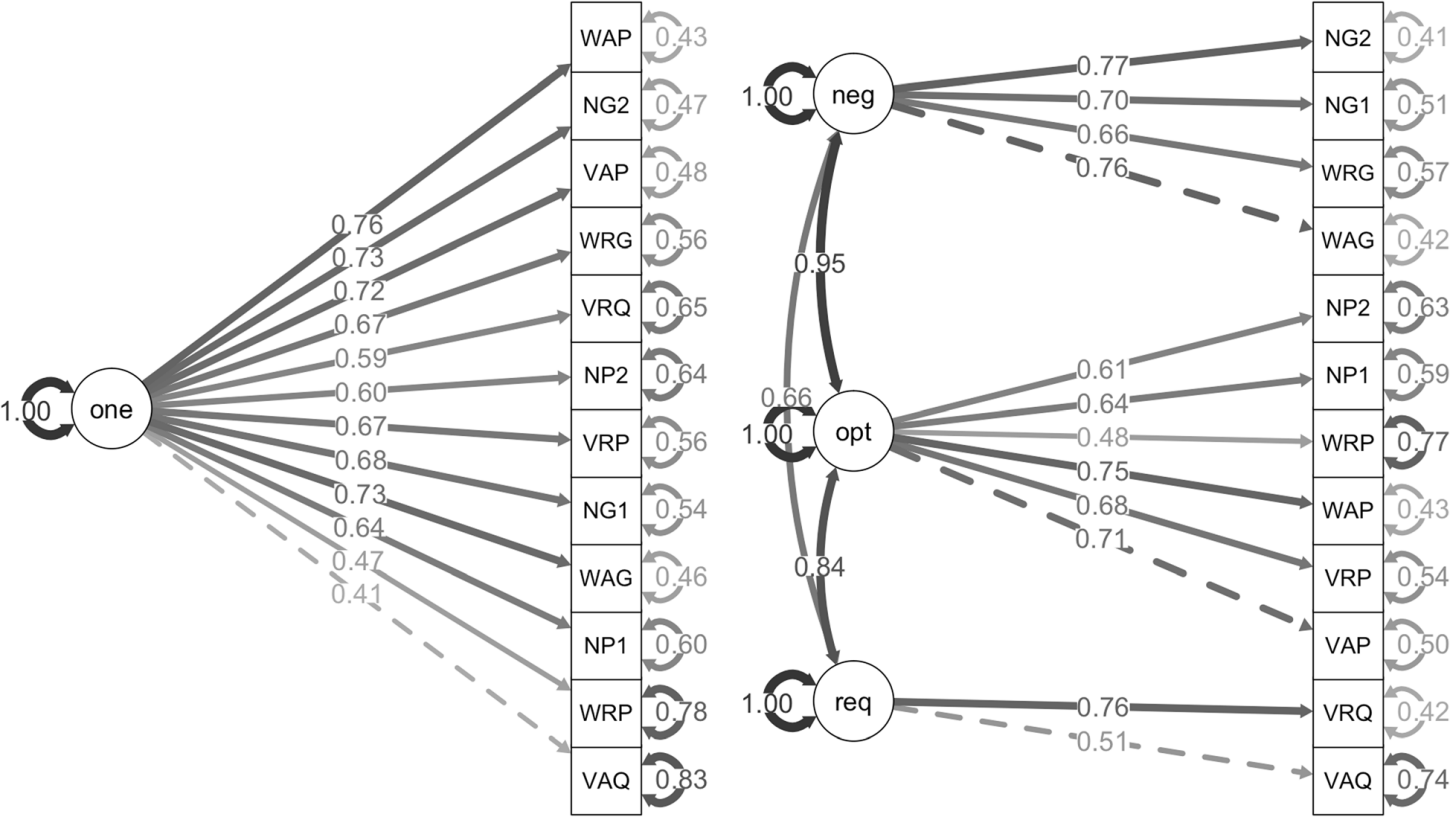
One-factor model (left) and level of spatial-thinking model (right) for ratings of teaching difficulty. One-factor model (one); negligible (neg), optional (opt), and required spatial thinking (req). The math problem were labeled first by type: visual (V), word (W), and notation (N); second by context: real-world (R), abstract (A), and notation (N); and third by level of spatial thinking: required (Q), optional (P), and negligible (G). Since notation types were also notation contexts, we balanced the number of problems by including two of each of these problems (indicated with a 1 and 2)

For ratings of teaching difficulty, the problem type and problem context models could not run due to “not positive definite” errors. This error indicates that there are zero or negative eigenvalues arising from linear dependency between the factors. The only way to fix these errors was to combine notation with word problem types and with abstract contexts. This reclassification of the math problems does not align with our categorization, and these reclassified models did not significantly outperform the one-factor model, so these models are not ideal for modeling this data. In contrast, the level of spatial-thinking model did outperform the one-factor model, *X*^*2*^(3) = 19.75, *p* < .001 (Fig. 4). This provides evidence that our categorization by level of spatial thinking is a good fit for this data.

**Fig. 5:**
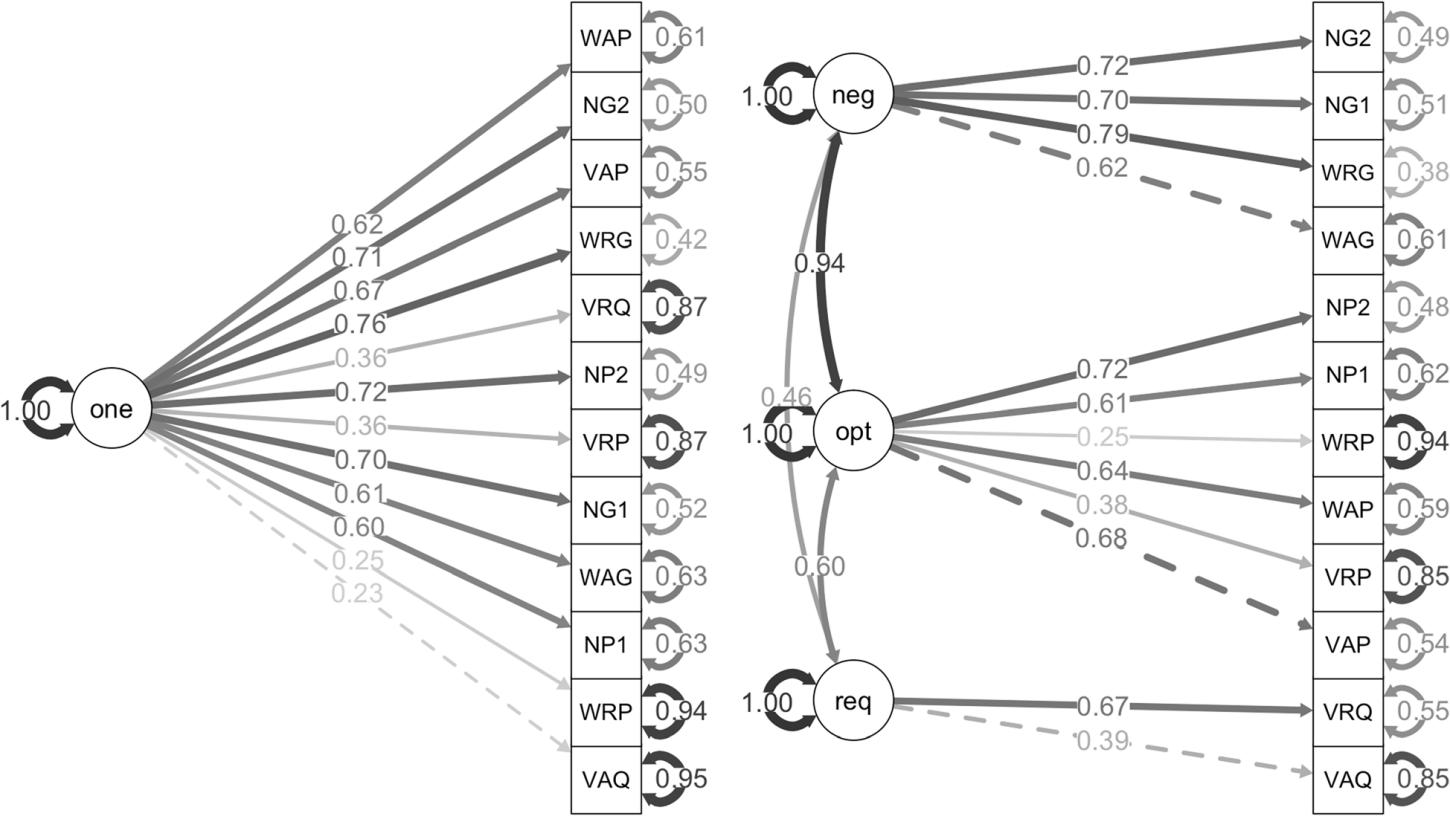
One-factor model (left) and level of spatial-thinking model (right) for ratings of spatial-thinking involvement. One-factor model (one); negligible (neg), optional (opt), and required spatial thinking (req). The math problem were labeled first by type: visual (V), word (W), and notation (N); second by context: real-world (R), abstract (A), and notation (N); and third by level of spatial thinking: required (Q), optional (P), and negligible (G). Since notation types were also notation contexts, we balanced the number of problems by including two of each of these problems (indicated with a 1 and 2)

**Fig. 6:**
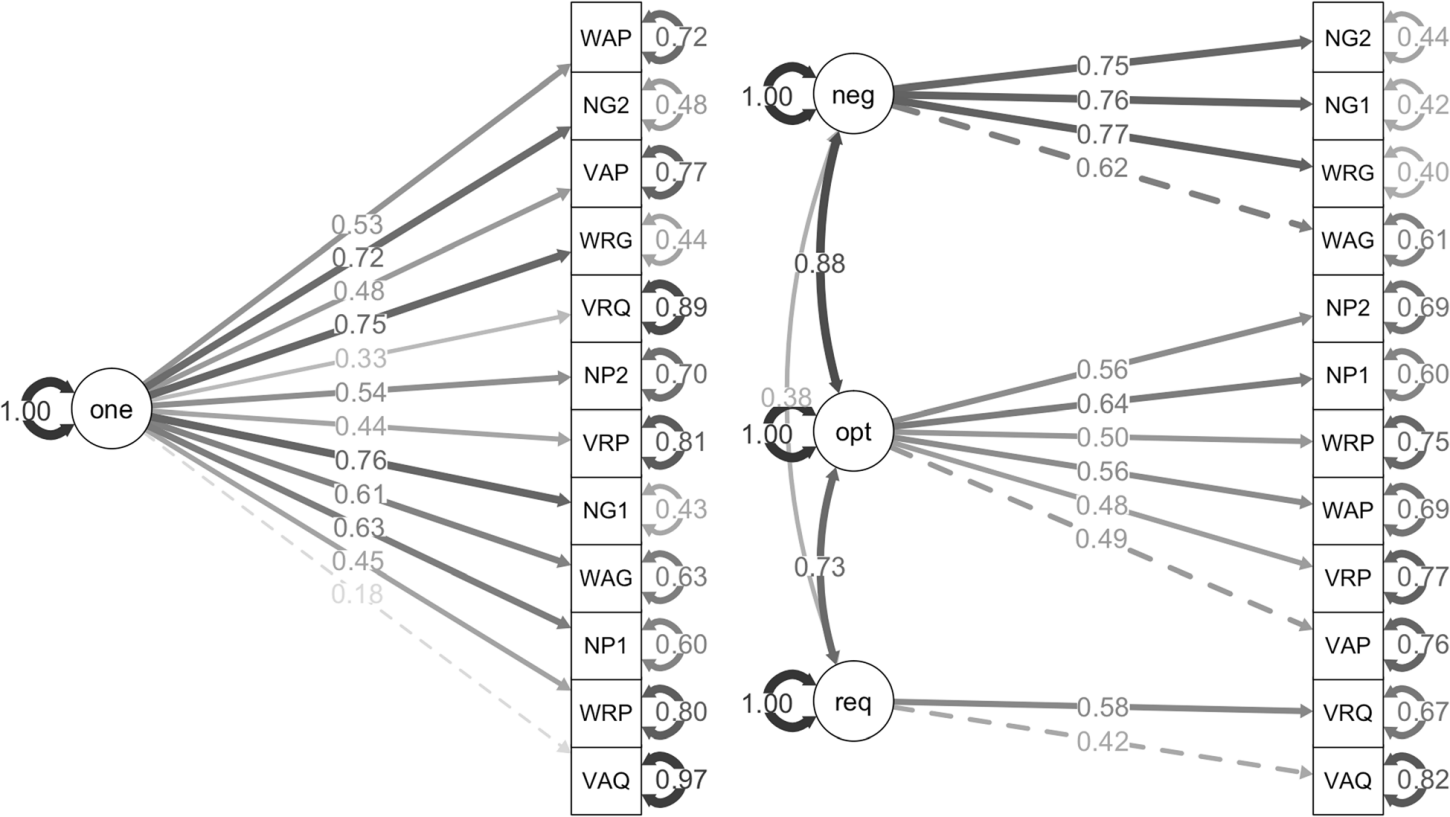
One-factor model (left) and level of spatial-thinking model (right) for ratings of visualization helpfulness. One-factor model (one); negligible (neg), optional (opt), and required spatial thinking (req). The math problem were labeled first by type: visual (V), word (W), and notation (N); second by context: real-world (R), abstract (A), and notation (N); and third by level of spatial thinking: required (Q), optional (P), and negligible (G). Since notation types were also notation contexts, we balanced the number of problems by including two of each of these problems (indicated with a 1 and 2)

A similar pattern was found with ratings of spatial-thinking involvement and ratings of the helpfulness of visualizations. The problem type and context models had “not positive definite” errors that could only be solved by reclassifying the math problems. The reclassified models did not significantly outperform the one-factor models. However, the level of spatial-thinking model did outperform the one-factor model for ratings of spatial-thinking involvement, *X*^*2*^(3) = 12.66, *p* < .01 (Fig. 5), and for ratings of the helpfulness of visualizations, *X*^*2*^(3) = 27.50, *p* < .001 (Fig. 6).

In summary, the structural equation models indicate that a one-factor model of all of the math problems together is not the best model of any of the three teacher ratings. Our categorization using problem type and problem context produced errors in the models, indicating that these two categories are not well suited for these teacher ratings. Finally, our categorization by spatial thinking was the best model and outperformed the one-factor model. Despite teachers rating math questions that differed by grade, Common Core Math standards, and specifics of the question, categorizing math problems by level of spatial thinking involved provided the best model of the data across the three ratings.

## Discussion

Student math success has many influences, including some related to their teachers. Teachers’ preference, knowledge-base, and attitudes toward math can impact their students’ math success. Further, spatial-thinking abilities relate to math success. The present work takes a first-step exploration of how teachers’ perceptions of their own spatial abilities (generally and as applied to math) might affect student math learning.

Elementary teachers completed multiple online surveys that together allow us to explore how self-reported spatial attitudes and ability relate to teaching/learning math, attitudes about teaching math, and experiences with the spatial aspects of math. Our results largely support relationships between teachers’ perceived spatial abilities and spatial attitudes and their math teaching. They also reinforced previous findings that math attitudes relate to math teaching.

### Connecting mathematics and spatial-thinking attitudes

Teachers rated spatial attitudes two ways, through competency and anxiety. Interestingly, these measures were not mirror-image perceptions. Teachers felt moderately spatially competent, but only slightly spatially anxious. Spatial competency was most strongly related to math efficacy and competency with the spatial aspects of their last math course, but spatial anxiety was most strongly related to teaching-math anxiety and NCTM alignment. The factor analysis provided additional support for these findings, as spatial competency loaded on the same factor as math efficacy and competency with the spatial aspects in math. Additionally, spatial anxiety loaded on the same factor as math anxiety, and NCTM alignment. Given these different relationships, spatial competency and anxiety might not be different sides of the same coin.

While it would be expected for spatial anxiety and competency to load on the same factor, they instead loaded on different factors with separate math measures. This finding supports the predicted connection between spatial thinking and mathematics’ attitudes. The first factor with spatial anxiety, math anxiety, and NCTM alignment likely indicates that stereotypical views of math (and perhaps also spatial thinking) are related to anxiety. It is possible that stereotypical viewpoints about math and spatial thinking cause an individual to experience anxiety when engaging in math or spatial tasks. Future research should investigate this possibility. The fourth factor with spatial competency, math efficacy, and spatial competency with the spatial aspects of their last math course, demonstrates a positive connection between spatial thinking and math. An individual who is confident in their spatial abilities generally, is also likely to be confident in their abilities to apply spatial thinking to math, and to use those spatial and math skills to support their math teaching. These two factors show another link between spatial thinking and mathematics.

### Spatial thinking in mathematics

Several measures addressed teachers’ identification, attitudes, and competency with spatial thinking in mathematics. First, higher-level math courses tended to involve more spatial thinking and teachers who took these higher-level courses reported handling the spatial aspects better, having lower spatial anxiety, being more effective in teaching and learning math, and holding less stereotypical math beliefs (i.e., NCTM alignment). Second, teachers who reported being competent in the spatial aspects of their last math course were also more spatially competent generally, less spatially anxious, less anxious teaching math, and considered themselves more effective in learning and teaching mathematics. It is interesting that education level related to NCTM alignment (but not competency with the spatial aspects) and competency with the spatial aspects related to spatial and math measures (but not education level). This might suggest that teachers who are spatially and mathematically inclined tend to take and do well in higher-level math courses, but it is the experience of taking higher-level courses that allows teachers to overcome stereotypical math beliefs.

### Role of teaching experience

Teaching experience could be measured both by years of teaching and by how frequently teachers taught math (daily or not). Not surprisingly, the number of years teaching was associated with less anxiety teaching math, greater NCTM alignment, and higher efficacy in teaching and learning math. In contrast, teaching math daily was associated with lower spatial anxiety, lower math-teaching anxiety, and greater NCTM alignment compared to those who taught math less regularly. In other words, daily math teaching related to both positive spatial and math-teaching attitudes. For those teachers who taught daily, years spent teaching math related to lower math anxiety and greater math efficacy. In sum, longer and regular experience (or willingness to gain longer and regular experience) teaching math is associated primarily with more positive attitudes and emotional reactions to mathematics.

### Math categorization

Our secondary explorations focused on the math categorization. We developed the math categorization to gain insights into challenges in teaching the spatial aspects of math. To validate the categorization, teachers rated teaching difficulty, visualization helpfulness, and spatial thinking involved on math problems differing in problem type, problem context, and spatial thinking involved. Results suggest this categorization’s usefulness. Teachers rated visual problems as the most spatial problem type, real-world contexts as the most spatial problem context, and ratings corroborated how the categorization ranked spatial-thinking involvement. Problems rated as most spatial were rated as more difficult to teach, but also as having higher potential for using visualizations. The structural equation models indicated that teacher ratings were best modeled by our categorization of the level of spatial thinking involved in solving the problems. Future work could use the categorization to investigate how teachers’ spatial competency relates to their teaching approaches for math concepts involves spatial thinking. It could also serve as a guide for incorporating spatial approaches to solving math problems, including using visualizations and/or manipulatives.

## Recommendations

Two recommendations for future research emerged from this work. First, spatial-thinking interventions for elementary students’ math learning will need to include interventions for teachers as well. We found that teachers’ beliefs and attitudes towards spatial thinking and math seem to be more tightly intertwined than elementary students’ spatial and math performance (Mix et al., 2016). Scaling effective spatial interventions into classrooms will require teaching teachers how to think spatially and apply that spatial thinking to math problems. While there are many teachers who have higher levels of math education, high levels of confidence in their spatial abilities, and excelled in the spatial aspects of their last math course, there are also many teachers who struggle with spatial thinking, think stereotypically about math, and might struggle with implementing spatial interventions into their classrooms. Future research should investigate how teachers’ beliefs and attitudes towards spatial thinking and math impact the effectiveness of spatial interventions in the classroom.

Second, this research indicated a possible mechanism for the connection between spatial and math anxiety. Spatial and math anxiety were both related to stereotypical views of math. Holding stereotypical views on a particular topic might be increase the likelihood of holding stereotypical views on other topics. Holding stereotypical views might put additional pressure on the individual to perform or “disprove” those stereotypes when engaging with the stereotyped topic. For instance, if a woman holds a stereotypical view that men are better at math than women, they might feel anxiety when completing a math test or feel that they must do well to counteract that stereotype (i.e., stereotype threat). Another possibility is that approaching math in a stereotypical way – that there is only one solution and only one possible path to that correct solution – might reduce an individuals’ likelihood of using a range of strategies to solve a math problem. Similarly, approaching spatial thinking in a stereotypical way – that people with good spatial abilities know the “correct” way of solving spatial problems – might reduce a person’s desire to attempt spatial problems and spatial thinking might be anxiety provoking for them. Future research is needed to understand the causal relationship between anxiety and stereotypical thinking and how that relationship connects approaches to solving math and spatial problems.

Three recommendations for teachers emerge from the current research. First, we suggest that teachers learn more about the literature connecting spatial thinking in mathematics and try building their spatial competencies. Newcombe (2010) wrote an overview of the connection between spatial thinking and STEM learning, along the educational implications of this connection, which is geared towards teachers. Our findings extend the existing literature by showing that spatial competence relates to perceived efficacy in teaching math and with belief alignment with math standards. These findings suggest there are benefits to teachers for focusing on both their own spatial thinking and spatial thinking in the classroom. The National Research Council (2006) supported this sentiment, noting that despite the importance of spatial training, it was missing in elementary education. The current work extends this suggestion to teachers.

Relatedly, we suggest that teachers focus on their own spatial competency, rather than spatial anxiety. Teachers rated themselves as moderately spatially competent but only slightly spatially anxious. The spatial competency scale may elicit more honest self-ratings of spatial-thinking capabilities, without the negative stigma of anxiety. These recommendations might also be pertinent for researchers.

Second, we suggest that teachers highlight spatial aspects of math to students. While teachers rated problems involving spatial thinking as more difficult to teach, relatively high spatial competency ratings suggest that teachers would succeed in teaching such problems. Further, giving students regular practice with spatial aspects of math may help build this core cognitive skill. This could, in turn, lead to greater success in more advanced math courses, which are perceived as having greater spatial content. Our math categorization may help teachers identify spatial thinking in math, potentially providing insight into why formulas work or providing additional solution paths. The categorization gives some guidance for lesson plans, focusing on general properties underlying problem solutions (i.e., spatial thinking involved, helpfulness of visualizations) and problem presentation (i.e., problem type and problem context). For instance, teachers could start math lessons using visual real-world problems that require spatial thinking. These problems would get students creating or interpreting visualizations that are relatable to their real-world experience, while also engaging their spatial thinking.

Third, even teachers who are anxious about doing or teaching math should pursue additional math experiences (courses, workshops, or professional development). These additional experiences may contribute to a better alignment between a teachers’ beliefs and the NCTM standards, might improve teachers’ perception of their math efficacy, and potentially improve their students’ math learning. Teaching math daily reduces math-teaching anxiety, which in turn helps teachers develop effective math-teaching methods. Additional math experiences may also challenge math stereotypes, such as the idea that there are “math people.” Teachers who hold such stereotypes and have math anxiety have a strong impact on their student’s math performance (Anderson, Boaler, & Dieckmann, 2018). In contrast, challenging these views positively impacted on how teachers taught math, their students’ beliefs about math, and their student’s math score (Ramirez, Hooper, Kerting, Ferguson, & Yeager, 2018).

## Conclusion

In conclusion, our findings provide a first step in establishing a connection between teachers’ spatial competency/anxiety and their math teaching. This extends previous findings on teachers’ math anxiety and teaching. In this research, teachers’ spatial competency/anxiety and competency with spatial aspects of math both related to math-teaching anxiety, math beliefs, and efficacy in teaching and learning math. Our math categorization may help identify when spatial thinking arises in math, cuing teachers to highlight spatial solutions. Because mathematical reasoning and spatial thinking share many points of connection, it is important for teachers to foster this connection in the classroom.

## Notes

In the structural equation models, the modeling of separate levels of spatial thinking was indicated as negligible (neg), optional (opt), and required spatial thinking (req). Further, the categorization of the math problems was indicated using a three-digit code. Problem type included visual (V), word (W), and notation (N). Problem context included real-world (R), abstract (A), and notation (N). Level of spatial thinking included required (Q), optional (P), and negligible (G). Since notation types were also notation contexts, we balanced the number of problems by including two of each of these problems (indicated with a 1 and 2).

## Data Availability

The dataset analyzed in this study is available from the corresponding author on reasonable request.

## Abbreviations

NCTM: National Council of Teachers of Mathematics standards
STEM: Science, Technology, Engineering, and Mathematics
CFI: Comparative Fit Index
TLI: Tucker Lewis Index
RMSEA: Root Mean Square Error of Approximation
SRMR: Standardized Root Mean Square Residual
AIC: Akaike Information Criterion
ECVI: Expected Cross Validation Index

## References

[CR1] Anderson RKA, Boaler J, Dieckmann JA (2018). Achieving elusive teacher change though challenging myths about learning: A blended approach. Education Sciences.

[CR2] Ashcraft MH (2002). Math anxiety: Personal, educational, and cognitive consequences. Current Directions in Psychological Science.

[CR3] Ball DL (1990). The mathematical understandings that prospective teachers bring to teacher education. Elementary School Journal.

[CR4] Ball DL, Hill HC, Bass H (2005). Knowing mathematics for teaching. American Educator.

[CR5] Beilock SL, Gunderson EA, Ramirez G, Levine SC (2010). Female teachers’ math anxiety affects girls’ math achievement. Proceedings of the National Academy of Sciences.

[CR6] Bekdemir M (2010). The pre-service teachers’ mathematics anxiety related to depth of negative experiences in mathematics classroom while they were students. Educational Studies in Mathematics.

[CR7] Brady P, Bowd A (2005). Mathematics anxiety, prior experience and confidence to teach mathematics among pre-service education students. Teachers and Teaching: Theory and Practice.

[CR8] Bursal M, Paznokas L (2006). Mathematics anxiety and preservice elementary teachers’ confidence to teach mathematics and science. School Science and Mathematics.

[CR9] Burte, H., Gardony, A. L., Hutton, A., & Taylor, H. A. (2017). Think3d!: Improving mathematics learning through embodied spatial training. *Cognitive Research: Principles and Implications*, *2*(13). 10.1186/s41235-017-0052-9. 10.1186/s41235-017-0052-9 PMC5318486 28275706

[CR10] Casey MB, Nuttall RL, Pezaris E (1997). Mediators of gender differences in mathematics college entrance test scores: a comparison of spatial skills with internalized beliefs and anxieties. Developmental Psychology.

[CR11] Cheng YL, Mix KS (2014). Spatial training improves children’s mathematics ability. Journal of Cognition and Development.

[CR12] Cheung, C. N., Sung, J. Y., & Lourenco, S. F. (2019). Does training mental rotation transfer to gains in mathematical competence? Assessment of an at-home visuospatial intervention. *Psychological Research*, 1–18. 10.1007/s00426-019-01202-5. 10.1007/s00426-019-01202-5 31144028

[CR13] Cornell C (1999). I hate math! I couldn’t learn it, and I can’t teach it!. Childhood Education.

[CR14] Ferguson AM, Maloney EA, Fugelsang J, Risko EF (2015). On the relation between math and spatial ability: The case of math anxiety. Learning and Individual Differences.

[CR15] Foley AE, Herts JB, Borgonovi F, Guerriero S, Levine SC, Beilock SL (2017). The math anxiety-performance link: A global phenomenon. Current Directions in Psychological Science.

[CR16] Ganley CM, Vasilyeva M (2011). Sex differences in the relation between math performance, spatial skills, and attitudes. Journal of Applied Developmental Psychology.

[CR17] Geary DC, Saults SJ, Liu F, Hoard MK (2000). Sex differences in spatial cognition, computational fluency, and arithmetical reasoning. Journal of Experimental Child Psychology.

[CR18] Gunderson EA, Ramirez G, Beilock SL, Levine SC (2012). The relation between spatial skill and early number knowledge: the role of the linear number line. Developmental Psychology.

[CR19] Gunderson EA, Ramirez G, Beilock SL, Levine SC (2013). Teachers’ spatial anxiety relates to 1st-and 2nd-graders’ spatial learning. Mind, Brain, and Education.

[CR20] Gunderson EA, Ramirez G, Levine SC, Beilock SL (2012). The role of parents and teachers in the development of gender-related math attitudes. Sex Roles.

[CR21] Hadley KM, Dorward J (2011). The relationship among elementary teachers’ mathematics anxiety, mathematics instructional practices, and student mathematics achievement. Journal of Curriculum & Instruction.

[CR22] Harper NW, Daane CJ (1998). Causes and reduction of math anxiety in preservice elementary teachers. Action in Teacher Education.

[CR23] Hart LC (2002). Preservice teachers’ beliefs and practice after participating in an integrated content/methods course. School Science and Mathematics.

[CR24] Hegarty M, Montello DR, Richardson AE, Ishikawa T, Lovelace K (2006). Spatial abilities at different scales: Individual differences in aptitude-test performance and spatial-layout learning. Intelligence.

[CR25] Hill HC, Blunk ML, Charalambous CY, Lewis JM, Phelps GC, Sleep L, Ball JM (2008). Mathematical knowledge for teaching and the mathematical quality of instruction: An exploratory study. Cognition and Instruction.

[CR26] Hill HC, Rowan B, Ball DL (2005). Effects of teachers’ mathematical knowledge for teaching on student achievement. American Educational Research Journal.

[CR27] Ingersoll RM (1999). The problem of underqualified teachers in American secondary schools. Educational Researcher.

[CR28] Krisztián Á, Bernáth L, Gombos H, Vereczkei L (2015). Developing numerical ability in children with mathematical difficulties using origami. Perceptual and Motor Skills.

[CR29] Lauer JE, Lourenco SF (2016). Spatial processing in infancy predicts both spatial and mathematical aptitude in childhood. Psychological Science.

[CR30] Lawton CA (1994). Gender differences in way-finding strategies: Relationship to spatial ability and spatial anxiety. Sex Roles.

[CR31] Lowrie T, Logan T, Ramful A (2017). Visuospatial training improves elementary students’ mathematics performance. British Journal of Educational Psychology.

[CR32] Ma X (1999). A meta-analysis of the relationship between anxiety toward mathematics and achievement in mathematics. Journal for Research in Mathematics Education.

[CR33] Maloney EA, Waechter S, Risko EF, Fugelsang JA (2012). Reducing the sex difference in math anxiety: The role of spatial processing ability. Learning and Individual Differences.

[CR34] Mix KS, Cheng YL (2012). The relation between space and math: Developmental and educational implications. Advances in Child Development and Behavior.

[CR35] Mix KS, Levine SC, Cheng YL, Young C, Hambrick DZ, Ping R, Konstantopoulos S (2016). Separate but correlated: The latent structure of space and mathematics across development. Journal of Experimental Psychology: General.

[CR36] Mix KS, Levine SC, Cheng YL, Young CJ, Hambrick DZ, Konstantopoulos S (2017). The latent structure of spatial skills and mathematics: A replication of the two-factor model. Journal of Cognition and Development.

[CR37] National Council of Teachers of Mathematics (NCTM) (2000). Principles and standards for school mathematics.

[CR38] National Governors Association Center for Best Practices & Council of Chief State School Officers (2010). Common core state standards for Mathematics.

[CR39] National Research Council (2006). Learning to think spatially: GIS as a support system in the K-12 curriculum.

[CR40] Newcombe NS (2010). Picture this: Increasing math and science learning by improving spatial thinking. American Educator.

[CR41] Ramirez G, Hooper SY, Kerting NB, Ferguson R, Yeager D (2018). Teacher math anxiety relates to adolescent students’ math achievement. AERA Open.

[CR42] Swars SL, Daane CJ, Giesen J (2006). Mathematics anxiety and mathematics teacher efficacy: What is the relationship in elementary preservice teachers?. School Science and Mathematics.

[CR43] Taylor HA, Hutton A (2013). Think3d!: Training spatial thinking fundamental to STEM education. Cognition and Instruction.

[CR44] Uttal DH, Cohen CA (2012). Spatial thinking and STEM education: When, why, and how?. Psychology of Learning and Motivation – Advances in Research and Theory.

[CR45] Vukovic RK, Kieffer MJ, Bailey SP, Harari RR (2013). Mathematics anxiety in young children: Concurrent and longitudinal associations with mathematical performance. Contemporary Educational Psychology.

